# Actin depolymerization is able to increase plant resistance against pathogens via activation of salicylic acid signalling pathway

**DOI:** 10.1038/s41598-019-46465-5

**Published:** 2019-07-18

**Authors:** Hana Leontovyčová, Tetiana Kalachova, Lucie Trdá, Romana Pospíchalová, Lucie Lamparová, Petre I. Dobrev, Kateřina Malínská, Lenka Burketová, Olga Valentová, Martin Janda

**Affiliations:** 1 Laboratory of Plant Biochemistry, Department of Biochemistry and Microbiology, University of Chemistry and Technology Prague, Technicka 5, 166 28 Prague 6, Czech Republic; 2 Laboratory of Pathological Plant Physiology, Institute of Experimental Botany of The Czech Academy of Sciences, Rozvojova 263, 165 02 Prague 6, Czech Republic; 3 Laboratory of Hormonal Regulations in Plants, Institute of Experimental Botany of The Czech Academy of Sciences, Rozvojova 263, 165 02, Prague 6, Czech Republic; 4 Department of Biochemistry, Faculty of Science, Charles University in Prague, Faculty of Science, 128 44 Hlavova 2030/8, Prague 2, Czech Republic; 5 Present Address: Ludwig-Maximilians-University of Munich (LMU), Faculty of Biology, Biocenter, Department Genetics, Grosshaderner Str. 2-4, D-82152 Martinsried, Germany

**Keywords:** Plant hormones, Plant immunity

## Abstract

The integrity of the actin cytoskeleton is essential for plant immune signalling. Consequently, it is generally assumed that actin disruption reduces plant resistance to pathogen attack. Here, we demonstrate that actin depolymerization induced a dramatic increase in salicylic acid (SA) levels in *Arabidopsis thaliana*. Transcriptomic analysis showed that the SA pathway was activated due to the action of isochorismate synthase (ICS). The effect was also confirmed in *Brassica napus*. This raises the question of whether actin depolymerization could, under particular conditions, lead to increased resistance to pathogens. Thus, we explored the effect of pretreatment with actin-depolymerizing drugs on the resistance of *Arabidopsis thaliana* to the bacterial pathogen *Pseudomonas syringae*, and on the resistance of an important crop *Brassica napus* to its natural fungal pathogen *Leptosphaeria maculans*. In both pathosystems, actin depolymerization activated the SA pathway, leading to increased plant resistance. To our best knowledge, we herein provide the first direct evidence that disruption of the actin cytoskeleton can actually lead to increased plant resistance to pathogens, and that SA is crucial to this process.

## Introduction

The actin cytoskeleton plays a key role in plant immunity^1,2^, both by providing a physical barrier and by its involvement in the transport of callose, antimicrobial compounds and cell wall components to an infection site^3^. Additionally, actin filament reorganization is a very fast response to treatment with conserved microbial compounds, MAMPs (microbe-associated molecular patterns), such as flg22, elf26 and chitin. The recognition of MAMPs triggers a specific set of immune responses, including cytoskeleton reorganization. It underpins the important role of actin cytoskeleton in plant defense^4,5^. Several studies have shown that when drugs, such as cytochalasins or latrunculin B, depolymerize the actin cytoskeleton, different plant species become more susceptible to pathogens. For example, treatment of *A*. *thaliana* with latrunculin B resulted in higher susceptibility to infection by *Pseudomonas syringae*^5–7^. In plants, actin depolymerizing factors serve to sever filamentous actin. The *adf4* (Actin Depolymerizing Factor 4) *A*. *thaliana* knock out mutant had reduced resistance to *Pseudomonas syringae* pv *tomato DC 3000* (*Pst* DC3000) expressing the AvrPphB effector^8^. This is because ADF4 is necessary for the expression of RPS5, the resistance protein that recognises AvrPphB^9^. However, in the intact *adf4* mutant, the density and skewness of actin filaments were the same as in control plants, implying that the actin cytoskeleton is not modified before infection^10^. ADF4 plays an indispensable role in the actin reorganisation upon elf26, but not in response to chitin^4^. The importance of actin cytoskeleton is also highlighted by the fact that *Pst DC3000* secretes at least two effectors modulating actin cytoskeleton. The effector HopW1 disrupts the actin cytoskeleton^6,7^. Another effector, HopG1, was shown to affect the remodelling of the actin cytoskeleton in *Pst* DC3000-infected *A*. *thaliana*^11^. Furthermore, treatment with cytochalasin E increased the penetration of *A*. *thaliana* plants by *Colletotrichum* species^12^ and the rate of entry to barley by *Blumeria graminis* f. sp. *hordei*^13^. Non-host resistance to *Erysiphe pisi* decreased after treatment with cytochalasins in barley, wheat, cucumber and tobacoo^14^, as did resistance to *Blumeria graminis* f. sp. tritici after cytochalasin E treatment of *A*. *thaliana*. Moreover, treatment with cytochalasin E in the absence of EDS1 (enhanced diseased resistance 1), an upstream component of the salicylic acid (SA) signalling pathway, strongly enhanced the inhibitory effect on non-host resistance^15^. However, in tobacco, cytochalasin E induced the transcription of *NtPR-1* (pathogenesis-related 1), a defence-related SA marker gene, and is able to prime cells to HR-like cell death in response to *Erysiphe cichoracearum*^16^. Furthermore, both cytochalasin E and latrunculin B induced the transcription of several SA marker genes (*AtPR-1*, *AtPR-2* and *AtWRKY38*) in *A*. *thaliana* seedlings^17^. This suggests that while such drugs do indeed cause actin depolymerization, the effects of such depolymerization may not always be adverse. Could it be that drug-induced actin depolymerization actually triggers processes that induce the SA pathway and thereby increase plant resistance to pathogens?

## Results

### Actin depolymerization induce salicylic acid biosynthesis via ICS1 dependent pathway

**Figure 1:**
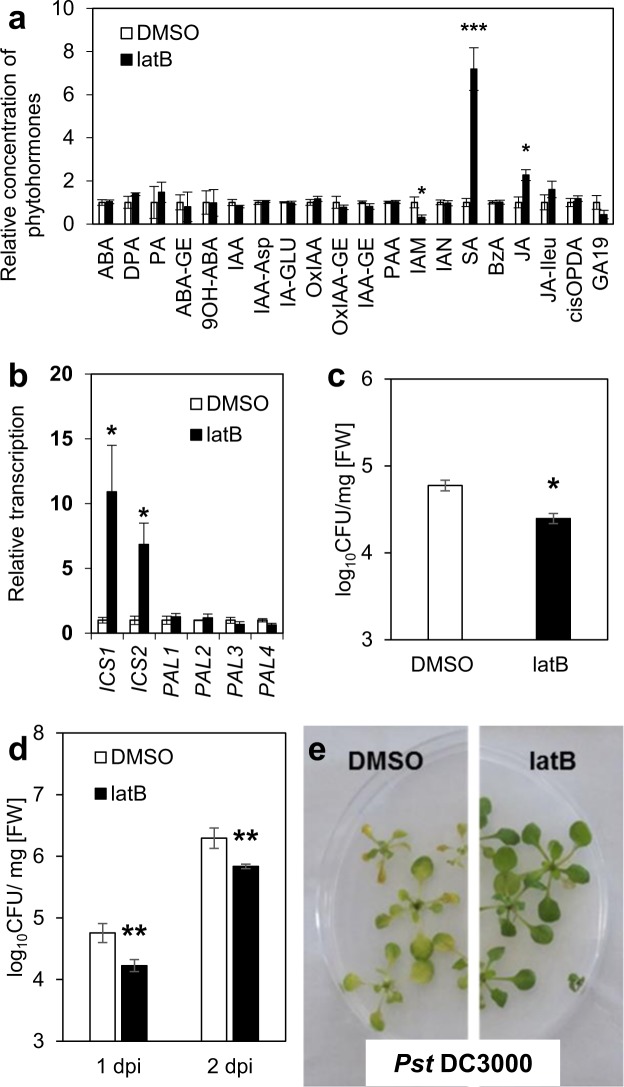
Seedlings of *A*. *thaliana*: Latrunculin B triggers SA biosynthesis and resistance to *Pst* DC3000. Seedlings were grown *in vitro* in liquid MS medium (**a**–**c**) and seedlings were grown *in vitro* in solid MS/2 medium (**d**,**e**). (**a**) Phytohormone analysis. Seedlings were treated for 24 h with 200 nM latrunculin B (latB) or 0.01% DMSO (control). For abbreviations of analyzed phytohormones, see Table S1. (**b**) Transcription of SA biosynthetic genes *ICS1*, *ICS2*, *PAL1*, *PAL2*, *PAL3* and *PAL4*. Seedlings were treated for 24 h with 200 nM latB or 0.01% DMSO. The transcription level was normalized to the reference gene, *SAND*. (**c**) Bacterial titres (liquid medium). Seedlings were pretreated for 24 h with 200 nM latB or 0.01% DMSO before inoculation with *Pst* DC3000. Tissue was harvested 1 day after inoculation with bacteria. (**d**) Bacterial titres (solid medium). Seedlings were pretreated for 24 h with 200 nM latB or 0.01% DMSO before inoculation with *Pst* DC3000. Tissue was harvested 1 and 2 days after inoculation with bacteria. (**e**) Representative photographs of seedlings grown on solid medium 2 days after inoculation with *Pst* DC3000. The values represent mean and error bars (SEM) from four (**a,c**), three to four (**b**) and five (**d**) independent samples. The asterisks represent statistically significant changes in latB-treated samples compared with controls (*P < 0.05; **P < 0.01; ***P < 0.001; two tailed Student’s t-test).

To establish that SA levels can increase upon actin depolymerization, we measured phytohormone content in *A*. *thaliana* seedlings treated with just 200 nM latrunculin B. Such a low concentration of latrunculin B proved sufficient to depolymerize actin filaments in the seedlings within 24 h (Fig. S1). Additionally, we showed that 24 h treatment with latrunculin B does not induce plant cell death (Fig. S2). Significantly, by that time there was a sevenfold increase in the free SA level of the treated seedlings compared with the control ones. The only other phytohormone to display an increase (twofold) was jasmonic acid (JA). Apart from Indole-3-acetamide (IAM), which showed a threefold decrease, the other tested phytohormones remained largely unaltered (Fig. 1a; Table S1).

Having shown this dramatic rise in SA level in *A*. *thaliana*, we wondered which of its two SA biosynthetic pathways was responsible for this increase or whether they both contributed to it. One pathway involves phenylalanine ammonia-lyase (PAL, EC 4.3.1.24), which exists in four isoforms, while the other involves isochorismate synthase (ICS; EC 5.4.4.2), which occurs in two isoforms^18^. Analysis of the transcription of all *AtPAL* and *AtICS* genes in the seedlings revealed that only the *AtICS* genes were induced by latruculin B (Fig. 1b). This shows that drug-induced actin depolymerization activates the ICS-dependent pathway and that this pathway alone is responsible for SA biosynthesis under these conditions.

### Actin depolymerization leads to induced resistance of *A*. *thaliana* against *Pst* DC3000

Given that increased resistance to pathogens in *A*. *thaliana* is associated with SA biosynthesis through the ICS pathway^19,20^, is it possible that activation of the same pathway invoked by drug-induced actin depolymerization also results in increased resistance? To investigate this, we used Ishiga *et al*.^21^ protocol as a basis for performing two *in vitro A*. *thaliana-Pseudomonas syringae* pv. *tomato* DC3000 (*Pst* DC3000) flood-inoculation assays in liquid and solid media^21^. We treated the seedlings with latrunculin B 24 h before inoculation with *Pst* DC3000. Remarkably, under both conditions, the latrunculin B-pretreated seedlings were more resistant than the control ones (Fig. 1c,d,e).

**Figure 2:**
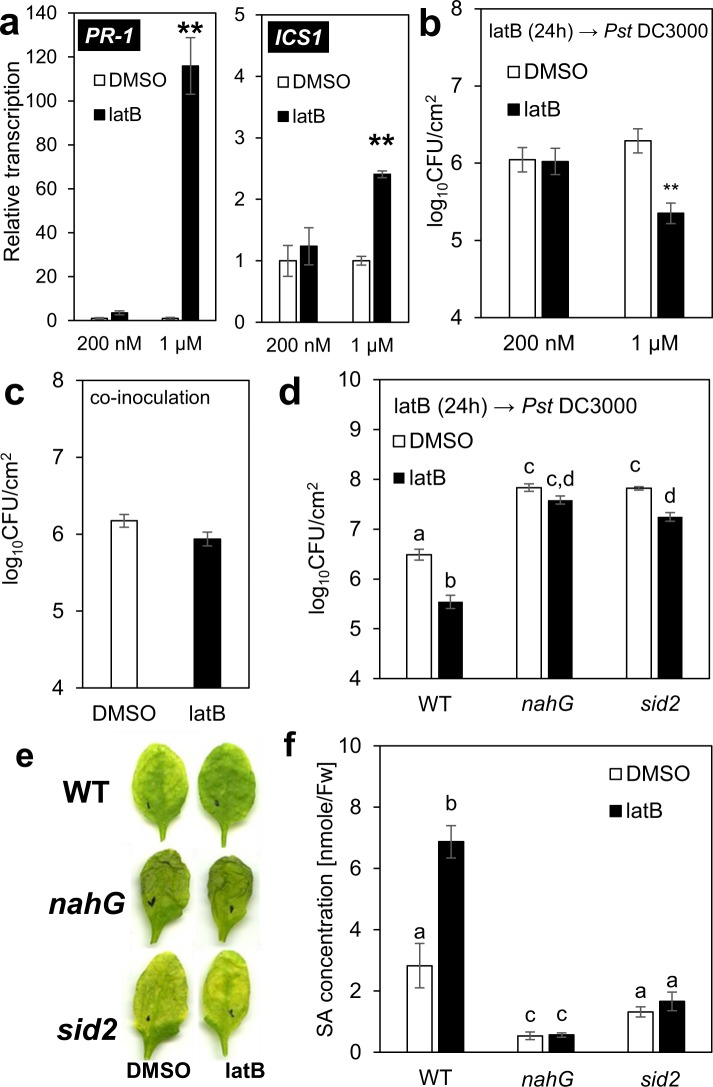
Four-week-old *A*. *thaliana*: Latruculin B-triggered SA pathway is necessary for higher resistance to *Pst* DC3000 (**a**). Transcription of SA marker genes *PR-1* and *ICS1* in four-week-old *A*. *thaliana* plants. Plants were treated for 24 h with 200 nM or 1 µM latrunculin B (latB). The transcription level was normalized to the reference gene, *TIP41*. (**b,c**) Bacterial titres in four-week-old plants. (**b**) Plants were pretreated with 200 nM or 1 µM latB for 24 h before inoculation with *Pst* DC3000. Control plants were pretreated with 0.01 or 0.05% DMSO. (**c**) Plants were treated with 1 µM latB or 0.05% DMSO, each in a solution containing *Pst* DC3000. (**d**) Bacterial titres in four-week-old plants. (**e**) Representative photographs of adult *A*. *thaliana* leaves infected with *Pst* DC3000 3 days after inoculation. (**f**) Salicylic acid (SA) concentration after 24 h 1 µM latB treatment. Plants were treated for 24 h with 1 µM latB or 0.05% DMSO before inoculation with *Pst* DC3000. *A*. *thaliana* WT plants (col-0) and mutants with impaired SA pathways (*nahG* and *sid2*) were used (d, e, f). Tissue was harvested 3 days after inoculation with *Pst* DC3000. The values represent mean and error bars (SEM) from four (**a,f**) and six (**b,c,e**) independent samples. The asterisks represent statistically significant changes in latB-treated samples compared with controls (**P < 0.01; two tailed Student’s t-test) and statistical differences between the samples (**d,f**) were assessed using a one-way ANOVA, with a Tukey honestly significant difference (HSD) multiple mean comparison post hoc test. Different letters indicate a significant difference, Tukey HSD, P < 0.01, n = 6.

To ensure that this phenomenon is not just associated with *in vitro* conditions, we also performed experiments using four-week-old *A*. *thaliana* plants cultivated in soil, such plants typically being used for studies of *A*. *thaliana* resistance to *Pst* DC3000^22^. Unlike in the seedlings, 24 h treatment with 200 nM latrunculin B did not activate the SA pathway in the adult plants and, thus, no increased resistance was observed (Fig. 2a,b). However, the transcription of SA marker genes (*AtPR-1*, *AtICS1*) was induced after 24 h treatment with 1 µM latrunculin B (Fig. 2a), leading to increased resistance to *Pst* DC3000 (Fig. 2b). This suggests that plant resistance is strongly dependent on latrunculin B concentration, probably due to differences between the efficiency of latrunculin B-induced actin depolymerization in seedlings and in adult plants (Figs S1 and S3). Similar to latrunculin B, pretreatment with cytochalasin E led to both SA-induced gene transcription (Fig. S4a) and increased plant resistance to *Pst* DC3000 (Fig. S4b), thereby strengthening the notion that such resistance is due to the depolymerizing activity of cytoskeletal drugs. It should be noted that we exclude the antibacterial effect of latrunculin B because *Pst* DC3000 grew *in vitro* in the presence of latrunculin B at a similar rate as in the control medium (Fig. S5).

### The induced resistance caused by actin depolymerization is dependent on salicylic acid

To further demonstrate the dependence of such resistance on the SA pathway, we performed assays using mutants known to have an impaired SA pathway and thus be more susceptible to *Pst* DC3000: *nahG*, which induces low endogenous SA levels through the expression of SA-hydroxylase^23^, and *sid2*, a knock-out mutant of the *AtICS1* gene^24^. As expected, latrunculin B did not induce resistance in the *nahG* plants (Fig. 2d,e). *Sid2* plants treated with latrunculin B were more resistant compared to *sid2* controls. However, latrunculin B treated *sid2* plants were still more susceptible than WT controls (Fig. 2e). The SA level is not induced in *sid2* plants (Fig. 2f) which correlates with the fact that none of SA biosynthetic genes does have induced transcription (Fig. S6b). Contrarily in seedlings, *ICS2* transcription is induced by latB (Fig. S6a). Altogether these results clearly confirm the crucial role of SA for actin depolymerization-induced resistance. However, increased resistance of latB treated *sid2* mutants uncover a new possible unknown SA independent mechanism triggering immunity.

### Actin depolymerization induce SA pathway in *B*. *napus* and enhance its resistance against *L*. *maculans*

**Figure 3:**
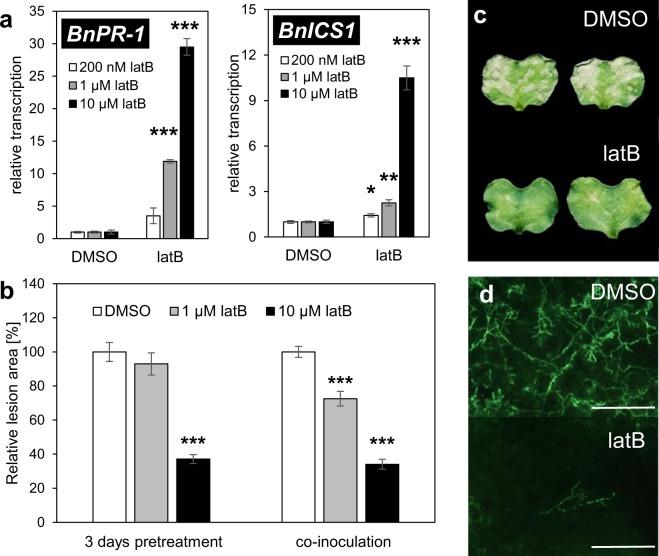
*B. napus* cotyledons: Latrunculin B triggers SA pathway and resistance to *L. maculans* (**a**). Transcription of SA marker genes *BnPR-1* and *BnICS1* in *B*. *napus* cotyledons. Cotyledons were treated for 24 h with infiltrations of 0.2, 1 or 10 µM latrunculin B (latB). Control cotyledons were treated for 24 h with a corresponding concentration of DMSO (0.01, 0.05 or 0.5%). The transcription level was normalized to the reference gene, *BnTIP41*. (**b**) *B*. *napus* susceptibility to *L*. *maculans* was evaluated as the relative lesion area (ratio of lesion area to whole leaf area) on the cotyledons. Cotyledons were treated with latrunculin B (latB; 1 µM or 10 µM) or DMSO control (0.05 or 0.5%), either 3 days before inoculation or simultaneously to inoculation by *L*. *maculans*. Lesions of DMSO controls in each treatment conditions were set as 100%. (**c**) Representative images of *L*. *maculans*-infected cotyledons. (**d**) Representative microscopy images of *L*. *maculans* hyphae proliferation in *B*. *napus* cotyledons in response to 10 µM latB or 0.5% DMSO. The bars correspond to 500 µM. The values represent mean and error bars (SEM) from three to four (**a**) and 60 -142 (**b**) independent samples. The asterisks represent statistically significant changes in latB-treated samples compared with controls (*P < 0.05; **P < 0.01; ***P < 0.001; two tailed Student’s t-test).

To show that this phenomenon is neither species-specific nor pathogen-specific, we investigated the effect of latrunculin B on an important crop, oilseed rape (*Brassica napus*). As in the case of *A*. *thaliana* in *B*. *napus*, latrunculin B upregulated the transcription of SA marker genes (*BnPR-1*, *BnICS1*) (Fig. 3a). Furthermore, as with adult *A*. *thaliana*, the effect of latrunculin B on *B*. *napus* was concentration dependent (Fig. 3a). The increased transcription of *BnPR-1* also occurred 72 h after latrunculin B treatment. On the other hand, *BnICS1* was not induced, indicating a transient effect of actin depolymerisation on *BnICS1* transcription (Fig. S7). The treatment of *B*. *napus* with 10 µM latrunculin B 3 days before inoculation with a hemibiotrophic fungal pathogen, *L. maculans*, efficiently inhibited hyphal colonisation and necrosis formation in the infected cotyledons (Fig. 3b,c,d). Treatment with 1 µM latrunculin B led to much weaker and variable resistance against *L*. *maculans* (Fig. 3b), corresponding to the weaker transcription of defence-related genes (Fig. 3a). These data are in accordance with our previous study characterizing the importance of SA in the defence of *B*. *napus* against *L*. *maculans*^25^. In addition, we observed significant cytochalasin E-induced resistance to *L*. *maculans* in *B*. *napus* (Fig. S8), which suggests that the effect is not compound-specific. Furthermore, neither latrunculin B nor cytochalasin E displayed antifungal activity on *L*. *maculans* growth *in vitro* (Fig. S9). Interestingly, the co-inoculation of *B*. *napus* cotyledons with a joint solution of 1 and 10 µM latrunculin B and *L*. *maculans* conidia also induced resistance (Fig. 3b).

## Discussion

### Our results indicate that depolymerized actin can trigger resistance to bacterial or fungal pathogens

**Figure 4:**
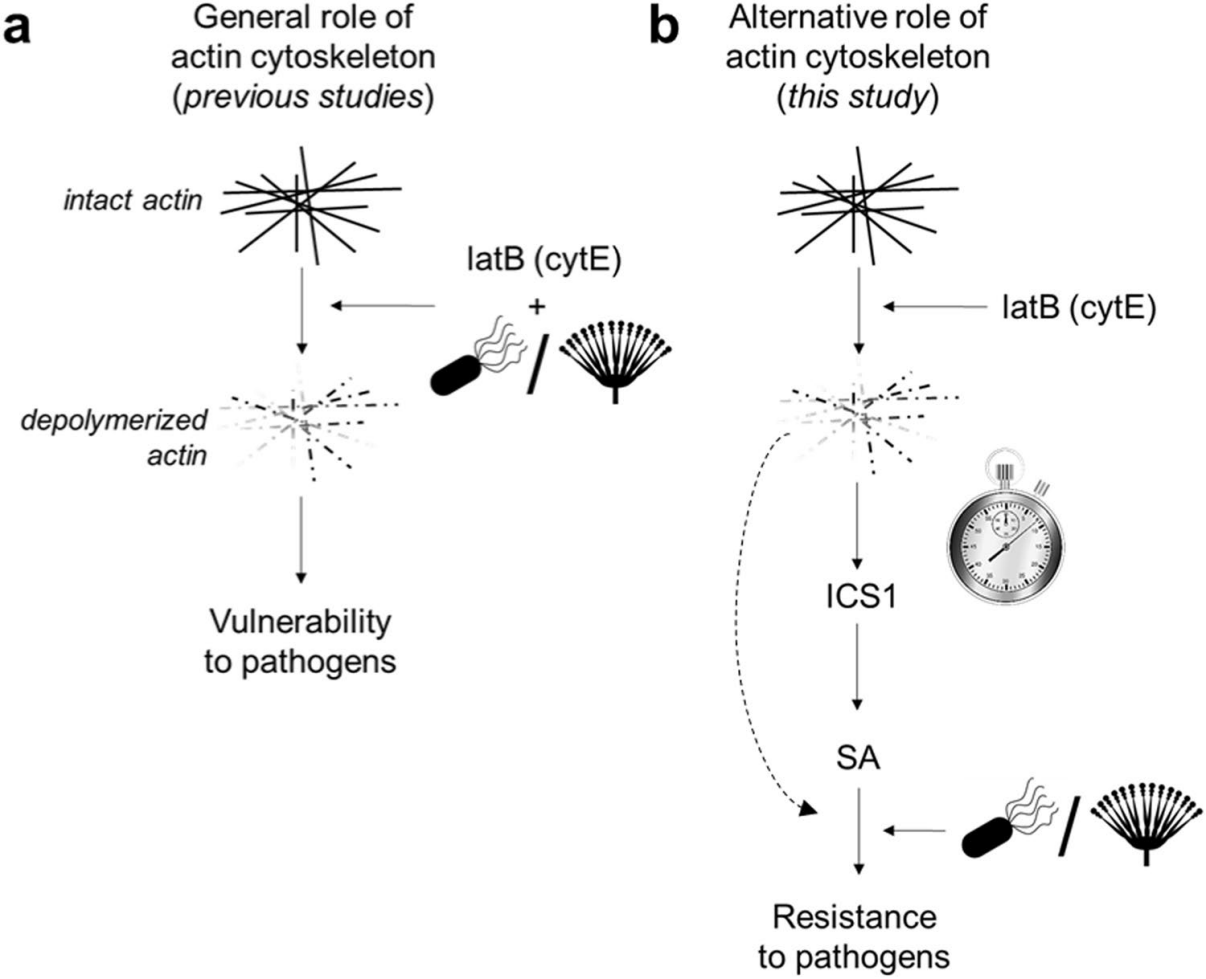
Possible dual role of actin cytoskeleton in plant response to pathogens. (**a**) The widely-published scenario in which depolymerization of the actin cytoskeleton by treatment with latrunculin B or cytochalasin E leads to increased plant vulnerability to pathogens. Studies showing this phenomenon co-inoculated plants with a drug and pathogen. (**b**) The new alternative scenario for the role of the actin cytoskeleton proposed in this manuscript. Plants pretreated with latrunculin B or cytochalasin E before inoculation with a pathogen have time to activate the salicylic acid signalling pathway, resulting in increased resistance to the subsequently inoculated pathogens. latB = latrunculin B; cytE = cytochalasin E; SA = salicylic acid; ICS1 = isochorismate synthase 1; = fungi;  = bacteria.

Thus, we have shown that plant immunity is strongly activated by depolymerised actin and that this phenomenon appears to be generally valid; namely, it seems not to be species specific, pathogen-type specific or drug-type specific. These findings do not negate those of previous studies that showed the susceptibility of plants treated with cytoskeletal drugs to pathogens^5–7,12,13,15^. Rather, they reveal that the plant disease resistance is strongly dependent on whether the plant has sufficient time to activate SA-mediated immunity (Fig. 4). This was clearly shown by our experiments with *Pst* DC3000, in which pre-infection treatment with cytoskeletal drugs resulted in resistance whilst co-inoculation did not (Fig. 2b,c,e). The co-inoculation of cytochalasin D and *Pst* DC3000 also had no effect on resistance according to Shimono *et al*.^11^. Other previous studies using actin-depolymerizing drugs showed higher susceptibility to *Pst* DC3000 when co-inoculation was used^5–7^(Fig. 4). It is also important to mention that actin filaments response to plant immunity is strongly dependent on conditions used in the study. A good example are effects of different MAMPs (flg22 and elf26) on actin reorganization. Using 24 day-old plants infiltrated with MAMPs, Henty-Ridilla *et al*.^5^ showed that treatment with flg22 induces actin reorganization, while elf26 does not^5^. Contradictorily to that, in epidermal cells of hypocotyl grown in the dark, Henty-Ridilla *et al*.^4^ showed that elf26 induces reorganisation and flg22 does not^4^ (an explanation could be that under these conditions, FLS2 receptor of flg22 is not expressed). In this study, we excluded the effect of different conditions on induced resistance of *A*. *thaliana* against *Pst DC3000* by testing three different setups (Figs 1c,d and 2b,e). The result was in all cases similar, whereby pretreatment with latrunculin B induced resistance of *A*. *thaliana* against *Pst DC3000*.

Interestingly, treatment with latrunculin B resulted in increased resistance in both *L*. *maculans* setups: pretreatment (Fig. 3b) and co-inoculation (Fig. 3b). This suggests that the rapidity of pathogen growth is a crucial factor. In contrast to *Pst* DC3000, which strongly damaged the inoculated leaves within three days, almost no multiplication of *L*. *maculans* occurred during the same period^25^. Thus, it appears that the slow growth of *L*. *maculans* enabled *B*. *napus* to establish the SA pathway, which was induced within 24 hours of cytoskeletal drug treatment (Fig. 3a). Overall then, while it is true that plant resistance to pathogens is decreased by a disrupted actin cytoskeleton, our results show that, given sufficient time, plants are able to trigger SA-based defence mechanisms to overcome such threats. This could be due to SA antimicrobial activity, accompanied by the SA-induced production of antimicrobial compounds. These powerful SA properties have been nicely demonstrated in relationship to so-called age-related resistance^26–28^. Our study shows that SA pathway, specifically induced by actin depolymerization, is more powerful despite the missing actin dynamics.

Up to date, some other works suggest the possible positive effect of depolymerization of actin cytoskeleton on plant immunity. Kobayashi and Kobayashi^16^ showed that treatment with cytochalins induce *NtPR1* transcription in tobacco. Additionally, cytochalasin E primed tobacco cells to induce HR-like cell death in presence of *Erysiphe cichoracearum*. We can speculate that it could lead to higher resistance against this biotrophic pathogen but it was not explicitly tested^16^. We confirmed the induction of *AtPR-1* gene upon treatment with cytoskeletal depolymerizing drugs in *A*. *thaliana*^17,29^. Recently it was shown that overexpression of *At*PRF3, which leads to depolymerization of actin filaments, increased ROS production upon flg22 treatment^30^. However to our best knowledge, we herein provide the first direct evidence that disruption of the actin cytoskeleton can actually lead to increased plant resistance to pathogens, and that SA is crucial to this process. We strongly believe that our work opens a new and important direction for further research. Is the influence of the actin cytoskeleton on vesicle trafficking involved in SA biosynthesis? For example, when PRRs (pattern recognising receptors) on the plasma membrane recognize MAMPs, it triggers PRRs endocytosis which, in turn, might activate the SA pathway^31,32^. A well characterised example is the internalization of FLS2, which is dependent on the actin-myosin complex^33^. Thus, could an imbalance in PRRs result in constitutively activated immunity and, thereby, induce SA biosynthesis? A hint in support of such a hypothesis is provided by a double mutant with impaired phosphatidylinositol-4-kinase β1 and β2 (*pi4kβ1β2*), which has been shown to alter vesicle trafficking and constitutively increase SA concentration^34,35^. It is also possible that plants have evolved a system for detecting actin cytoskeleton disruption and that the activation of such a system triggers SA-specific immune responses. However, as yet, we are not able to determine if chemically-depolymerised actin is really the triggering event for immune signalling or whether a pleiotrophic event, such as endoplasmic reticulum stress, results in SA induction. For this reason, further research should be focused on deciphering the specific mechanism by which actin depolymerization triggers SA biosynthesis and the ensuing increased plant resistance to pathogens.

## Materials and Methods

### Plant material

For the *A. thaliana* experiments, the following genotypes were used: Columbia-0 (WT); *sid2-3* (SALK_042603)^24^; *nahG*^23^ and *pUBC*::Lifeact-GFP^36^. *A*. *thaliana* seedlings were grown either in liquid MS medium or on solid MS/2 medium. Per litre, the liquid MS medium contained the following: 4.41 g Murashige and Skoog medium including vitamins (Duchefa, Netherlands), 5 g sucrose, 5 g MES monohydrate (Duchefa, Netherlands). Per litre, the solid MS/2 medium contained 2.2 g Murashige and Skoog medium (Duchefa, Netherlands) with 10 g sucrose and 8 g Plant agar (Duchefa, Netherlands). Both media were adjusted to pH 5.7 using 1 M KOH. For cultivation in the liquid, surface-sterilized seeds were sown in 24-well plates containing 400 μL of liquid MS medium per well. The plants were cultivated for 10 days under a short-day photoperiod (10 h/14 h light/dark regime) at 100–130 μE m^−2^ s^−1^ and 22 C. On the 7th day, the medium in the wells was exchanged for a fresh one. For cultivation on the solid MS/2 medium, seedlings were grown in Petri dishes for 12 days under a long-day photoperiod (16 h/8 h light/dark regime) at 100–130 μE m^−2^ s^−1^ and 22 °C. For *A*. *thaliana* plants grown for 4 weeks in soil, surface-sterilized seeds were sown in Jiffy 7 peat pellets and the plants cultivated under a short-day photoperiod (10 h/14 h light/dark regime) at 100–130 μE m^−2^ s^−1^, 22 °C and 70% relative humidity. They were watered with fertilizer-free distilled water as necessary

For the *B. napus* experiments, plants of the Eurol cultivar were grown hydroponically in perlite in Steiner’s nutrient solution (Steiner, 1984) under a 14 h/10 h light/dark regime (25 °C/22 °C) at 150 μE m^–2^ s^–1^ and 30–50% relative humidity. True leaves were removed from 14-day-old plantlets to avoid cotyledon senescence.

### Treatment with chemical compounds

As actin depolymerizing drugs, latrunculin B (Sigma-Aldrich, USA) and cytochalasin E (Sigma-Aldrich, USA) were used. Latrunculin B and cytochalasin E were both dissolved in DMSO; the concentration of the stock solutions were 2 mM and 4 mM, respectively.

For the *Pst* DC3000 resistance assay, the seedlings grown in 24-well plates were treated by replacing the pure liquid MS medium in the plate wells with medium containing 200 nM latrunculin B or 0.01% DMSO control. The seedlings cultivated on the solid medium were treated 24 h by flooding with 10 mL of MS/2 medium containing 200 nM latrunculin B or 0.01% DMSO control. Fully-developed leaves from four-week-old *A*. *thaliana* grown in soil were infiltrated either with 200 nM or 1 µM latrunculin B (0.01% or 0.05% DMSO as respective controls) or with 1 µM or 10 µM cytochalasin E (0.025% or 0.25% DMSO as respective controls) 24 h before *Pst* DC3000 infection using a needleless syringe.

For the transcriptomic assay, the seedlings *of A. thaliana* grown in 24-well plates were treated 24 h with 200 nM latrunculin B (0.01% DMSO control) or 10 µM cytochalasin E (0.25% DMSO control). Four-week old *A. thaliana* were infiltrated with 200 nM (0.01% DMSO) or 1 µM latrunculin B (0.05% DMSO) for 24 h. The 10-day-old cotyledons of *B*. *napus* were infiltrated either with 1 µM or 10 µM latrunculin B or with 10 µM cytochalasin E (in all cases with corresponding DMSO controls) using a needleless syringe. For infection assay 3 days before infection with *L*. *maculans*, for transcriptomic assay 24 and 72 h before harvesting tissue.

### Inoculation of *A*. *thaliana* seedlings with *Pst* DC3000

After the *A*. *thaliana* seedlings had been cultivated in 24-well plates in the liquid MS medium for 10 days, the cultivation medium was exchanged for one containing latrunculin B or cytochalasin E, and incubated for 24 h. On day 11, the medium was replaced with a bacterial suspension of *Pst* DC3000 in 10 mM MgCl_2_ (OD_600_ = 0.01). The seedlings were incubated in this bacterial suspension for 1 min. After incubation, the suspension was replaced with the liquid MS medium. On day 12, the seedlings were harvested, each sample taken containing all of the seedlings from three wells. The seedlings were then homogenized in tubes with 1 g of 1.3 mm silica beads using a FastPrep-24 instrument (MP Biomedicals, USA). The resulting homogenate was serially diluted and pipetted onto King B plates. The colonies were counted after 1–2 days of incubation at 28 °C.

The seedlings cultivated on solid medium were flooded with 200 nM latrunculin B solution in water on day 13. Control plants were treated with a corresponding solution of DMSO. On day 14, the solutions were replaced with a suspension of overnight culture of *Pst* DC3000 (OD_600_ = 0.01) containing 0.025% Silwet. Samples were harvested at 0, 1 and 2 dpi, with each sample containing the plants from five plates. The seedlings were homogenized in tubes with 1 g of 1.3 mm silica beads using a FastPrep-24 instrument (MP Biomedicals, USA). The resulting homogenate was serially diluted and pipetted onto LB plates containing rifampicin. The colonies were counted after 1–2 days of incubation at 28 °C.

### Inoculation of four-week-old *A*. *thaliana* with *Pst* DC3000

*Pst* DC3000 was grown overnight on King B agar plates at 28 °C, resuspended in 10 mM MgCl_2_, and diluted to an OD_600_ of 0.001. Using a needleless syringe, the bacterial suspension was infiltrated into three fully-developed leaves from one plant. After 3 days, the infected tissue was collected as cut leaf discs (one disc per leaf, 0.6-cm diameter); three leaf discs from one plant represent one sample. The discs were homogenized in tubes with 1 g of 1.3 mm silica beads using a FastPrep-24 instrument (MP Biomedicals, USA). The resulting homogenate was serially diluted and pipetted onto King B plates. The colonies were counted after 1–2 days of incubation at 28 °C.

### Inoculation of *B. napus* with *L. maculans*

*L*. *maculans* isolate v23.1.3^25,37^ was used to inoculate *B*. *napus*. After harvesting, conidia obtained according to Šašek *et al*.^25^ were washed once with distilled water, diluted to 10^8^ spores/ml, and stored at –20 °C for up to 6 months. The cotyledons of 14-day-old plants were infiltrated by conidial suspension (10^5^ conidia/ml), with at least 12 plants being used for each inoculation. The leaves were assessed for lesions 10 days after inoculation. The leaf area and the lesion areas therein were measured by image analysis using APS Assess 2.0 software (American Phytopathological Society, USA). The relative lesion area was then calculated as the ratio of lesion area to whole leaf area. For the microscopy studies, the cotyledons infected with GFP-tagged v23.1.3 isolate^38^ were observed at 10 dpi using a Leica DM5000 B microscope.

### Gene expression analysis

The whole seedlings from three independent wells were immediately frozen in liquid nitrogen. The tissue was homogenized in tubes with 1 g of 1.3 mm silica beads using a FastPrep-24 instrument (MP Biomedicals, USA). Total RNA was isolated using a Spectrum Plant Total RNA kit (Sigma-Aldrich, USA) and treated with a DNA-free kit (Ambion, USA). Subsequently, 1 μg of RNA was converted into cDNA with M-MLV RNase H^−^ Point Mutant reverse transcriptase (Promega Corp., USA) and an anchored oligo dT21 primer (Metabion, Germany). Gene expression was quantified by q-PCR using a LightCycler 480 SYBR Green I Master kit and LightCycler 480 (Roche, Switzerland). The PCR conditions were 95 °C for 10 min followed by 45 cycles of 95 °C for 10 s, 55 °C for 20 s, and 72 °C for 20 s. Melting curve analysis was then conducted. Relative expression was normalized to the housekeeping genes *AtSAND* and *BnTIP41*. Primers were designed using PerlPrimer v1.1.21^39^. A list of the analysed genes and primers is available in Table S2.

### Phytohormonal analysis

Hormone analysis was carried out on four samples, each of which contained all seedlings from six of the 24 wells or from the four-week-old *A*. *thaliana* three leaf discs from every single plant were sampled, three individual plants were sampled as one sample. Plant hormone levels were determined as described by^40^. Briefly, samples were homogenized in tubes with 1.3 mm silica beads using a FastPrep-24 instrument (MP Biomedicals, USA). The samples were then extracted with a methanol/H_2_O/formic acid (15:4:1, v:v:v) mixture, which was supplemented with stable isotope-labeled phytohormone internal standards (10 pmol per sample) in order to check recovery during purification and validate the quantification. The clarified supernatants were subjected to solid phase extraction using Oasis MCX cartridges (Waters Co., USA). The eluates were evaporated to dryness and the generated solids dissolved in 30 μl of 15% (v/v) acetonitrile in water. Quantification was performed on an Ultimate 3000 high-performance liquid chromatograph (Dionex, USA) coupled to a 3200 Q TRAP hybrid triple quadrupole/linear ion trap mass spectrometer (Applied Biosystems, USA) as described by^41^. Metabolite levels were expressed in pmol/g fresh weight (FW).

### Confocal microscopy of actin filaments

For *in vivo* microscopy, a Zeiss LSM 880 inverted confocal laser scanning microscope (Carl Zeiss AG, Germany) was used with either a 40× C-Apochromat objective (NA = 1.2 W) or a 20x Plan-Apochromat objective (NA = 0.8). GFP fluorescence (excitation 488 nm, emission 489–540 nm) was acquired in z-stacks (20–25 µm thickness). The maximum intensity projections obtained from the z-stacks were created using Zeiss ZEN Black software. Actin filaments density analysis was calculated by Fiji software (https://fiji.sc/)^42^ as the percent occupancy of GFP signal in each Maximum intensity projection. Image threshold was set to include all actin filaments and area fraction was measured. We analysed 7–11 cutouts from 6–7 plants for each variant. Representative images were selected from photos from at least 6 independent plants.

### Growth of *Pst* DC3000 and *L*. *maculans in vitro* in presence of latrunculin B or cytochalasin E

*Pst* DC3000 grew overnight on solid LB medium containing rifampicin. From this, a fresh bacterial suspension was prepared (OD_600_ = 0.01) in liquid LB or liquid MS medium. To this suspension, latrunculin B (200 nM or 1 µM) or DMSO (0.05% or 0.01%) was added. The OD_600_ was measured 6 and 24 h after suspension preparation. Four independent samples were prepared for each type of treatment.

Conidia of the GFP-tagged v23.1.3 isolate of *L*. *maculans*^38^ were grown *in vitro* in Gamborg B5 medium (Duchefa, Netherlands) supplemented with 0.3% (w/v) sucrose and 10 mM MES monohydrate, and adjusted to pH 6.8. This medium contained latrunculin B (1, 10 µM), cytochalasin E (1, 10 µM) or DMSO control (0.5%), and had a final concentration of 2500 conidia per well. The plates (black 96-well plate, Nunc R), covered with lids and sealed with Parafilm®, were incubated in darkness at 26 °C. On day 4, fluorescence was measured using a Tecan F200 fluorescence reader (Tecan, Switzerland) equipped with a 485/20 nm excitation filter and 535/25 nm emission filter. Eight wells were measured for each treatment.

### Trypan blue staining

Detached leaves were immersed to the staining solution (10 mL lactic acid (85%, w:w), 10 mL phenol, 10 mL glycerol, 10 mL dH_2_0, 40 mg trypan blue (final concentration 10 mg.mL^−1^) for 30 min due to Fernández-Bautista *et al*.^43^. Solution was then replaced by ethanol 3 times until leaves were fully decolored from chlorophyll. Leaves were rehydrated by replacing solution with the decreasing ethanol solutions (70%, 50%, 30%, v:v) and kept in water for the microscopy purposes.

### Statistical analyses

All experiments were repeated at least three times, except Fig. 3B where we put together data from 3–7 biological repetitions. All statistical analyses were performed with Microsoft Excel 2013. The P values were calculated using a two-tailed Student’s t-test or one-way ANOVA followed with Tukey honestly significant difference (HSD) p < 0,01 using software Statistica® v.11 or SigmaPlot11®.

## Supplementary information

Supplementary info
