# Supplementary material for: Actin depolymerization is able to increase plant resistance against pathogens via activation of salicylic acid signalling pathway
Source: Sci Rep. 2019 Jul 18;9:10397. doi: 10.1038/s41598-019-46465-5 (PMC6639534; doi:10.1038/s41598-019-46465-5)
Supplement: Supplementary file 1 — Supplementary info [file 41598_2019_46465_MOESM1_ESM.pdf]

SUPPLEMENTARY INFORMATION

Actin depolymerization is able to increase plant resistance against pathogens via activation of salicylic acid signalling pathway

Hana LEONTOVYČOVÁ[1,2,4], Lucie TRDÁ[2], Tetiana KALACHOVA[2], Lucie LAMPAROVÁ[1,2], Romana POSPÍCHALOVÁ[2], Petre I. DOBREV[3], Kateřina MALÍNSKÁ[3], Lenka BURKETOVÁ[2], Olga VALENTOVÁ[1], Martin JANDA[1,2,]

[1] Laboratory of Plant Biochemistry, Department of Biochemistry and Microbiology, University of Chemistry and Technology Prague, Technicka 5, 166 28 Prague 6, Czech Republic
[2] Laboratory of Pathological Plant Physiology, Institute of Experimental Botany of The Czech Academy of Sciences, Rozvojova 263, 165 02, Prague 6, Czech Republic
[3] Laboratory of Hormonal Regulations in PlantsInstitute of Experimental Botany of The Czech Academy of Sciences, Rozvojova 263, 165 02, Prague 6, Czech Republic
[4] Department of Biochemistry, Faculty of Science, Charles University in Prague, Faculty of Science, 128 44 Hlavova 2030/8, Prague 2, Czech Republic

FIGURE S1

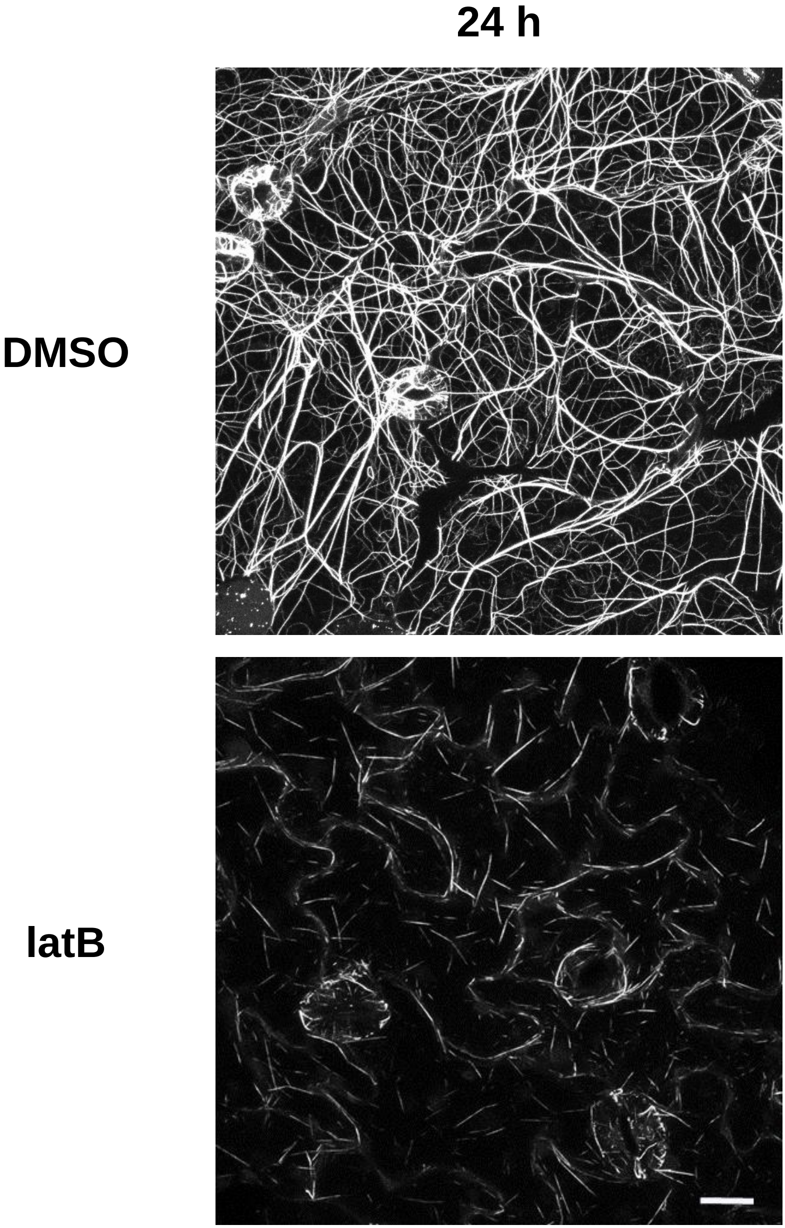

Fig. S1. Latrunculin B disrupts actin filaments in *A. thaliana* seedlings. Microscopy images (maximum intensity projections) of *pUBC::Lifeact-GFP A. thaliana* seedlings treated with 200 nM latrunculin B or 0.01% DMSO (control) for 24 h. The bar corresponds to 20 µm.

FIGURE S2

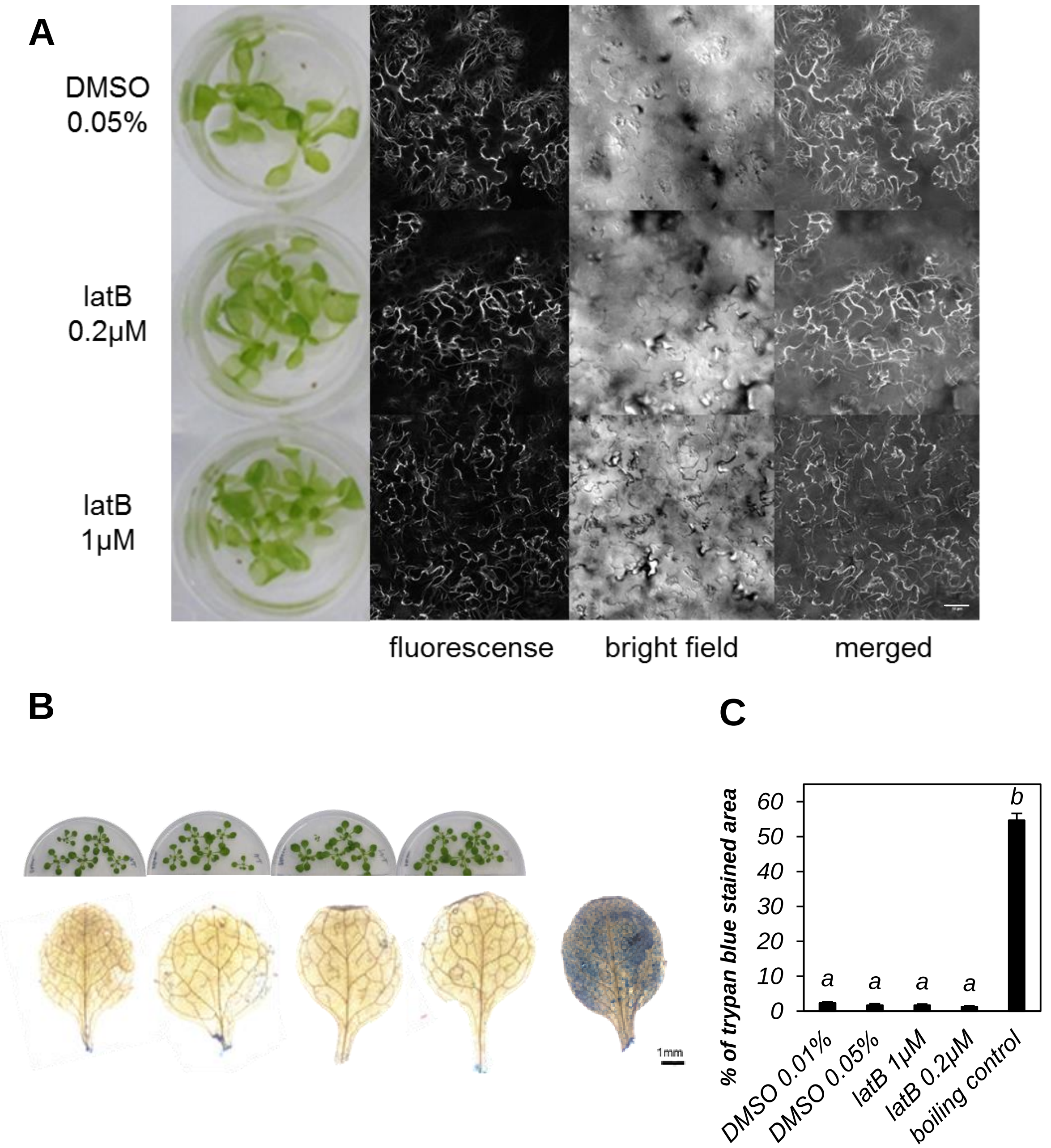

Fig. S2. Latrunculin B does not induce cell death in *A. thaliana* seedlings. A) Eleven days old seedlings of 35S::lifeAct-GFP were grown in MS liquid medium at 10h/14h light cycle and treated with latB for 24 h by replacing cultivation media with LanB-containing one; DMSO in appropriate concentrations was used as a control. Photos of the seedlings amd microscopic images of GFP-fluorescence and bright field channels. Scale bar corresponds to 10μm **B)** Fourteen days old seedlings grown on MS/2 agar plates at 10h/14h light cycle were treated with latB for 24h by flooding, DMSO with appropriate concentration and boiling of leaf tissues for 5 min at 100°C were used as controls. Photos of the seedling growth on plates after LanB treatment and images of trypan-blue stained leaf tissues; **C)** quantification of trypan blue stained area. Data are presented in means ±SE, different letters mean significant difference between treatments, two-taied *t*-test.

FIGURE S3

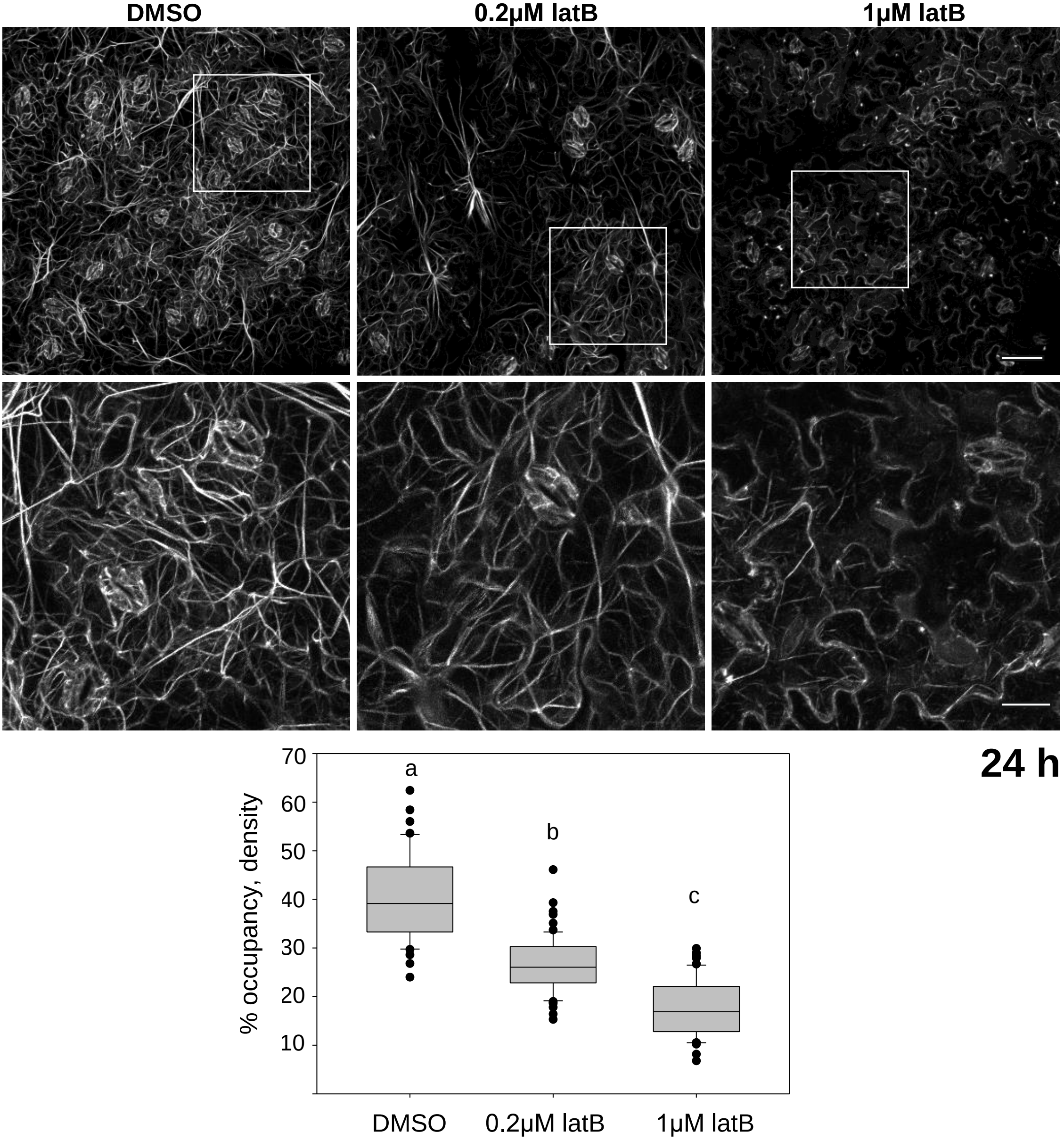

Fig. S3. Latrunculin B disrupts actin filaments in four-week-old *A. thaliana*. Microscopy images (maximum intensity projections) of *pUBC::Lifeact-GFP* four-week-old *A. thaliana* plants treated for 24 h with either latrunculin B (0.2 µM or 1 µM) or 0.05% DMSO (control). White squares in the upper row indicate borders of the presented details (lower row). The bars correspond to 50 µm and 20 µm, respectively. Degree of actin depolymerisation is quantified as the percent of occupancy of GFP signal in image. Statistical differences between the samples were assessed using a one-way ANOVA, with a Tukey honestly significant difference (HSD) multiple mean comparison post hoc test. Different letters indicate a significant difference, Tukey HSD, $P < 0.001$, n=43 to 72.

FIGURE S4

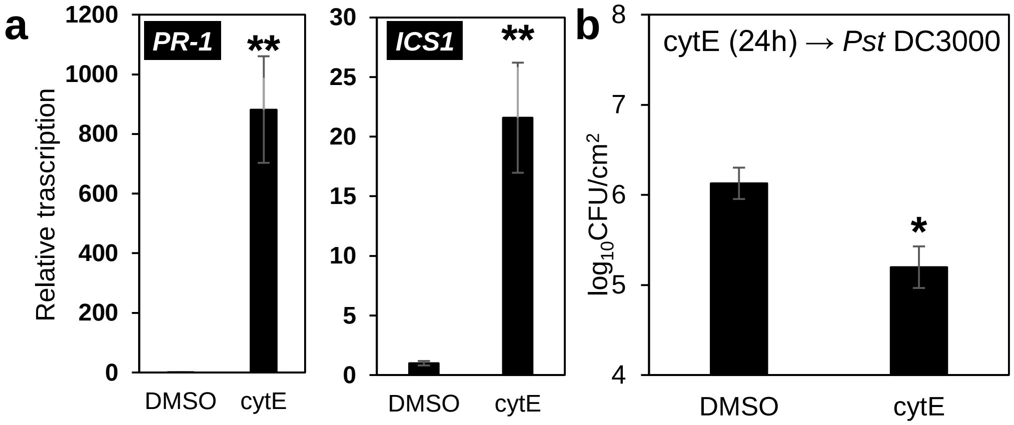

Fig. S3. Cytochalasin E induces SA-dependent gene transcription and resistance to *Pst* DC3000 in four-week-old *A. thaliana* plants. a) Transcription of SA marker genes *PR-1* and *ICS1* in four-week-old *A. thaliana* plants. Plants were treated for 24 h with 10 μM cytochalasin E (cytE). The transcription level was normalized to the reference gene, *TIP41*. **b)** Bacterial titres in four-week-old plants. Plants were pretreated with 10 μM cytE for 24 h before treatment with *Pst* DC3000. Control plants were treated with DMSO (0.25%). Tissue was harvested 3 days after inoculation with *Pst* DC3000. The values represent mean and error bars (SEM) from four independent samples. The asterisks represent statistically significant changes in the cytE-treated samples compared with controls (* P<0.05; ** P<0.01; two tailed Student´s t-test).

FIGURE S5

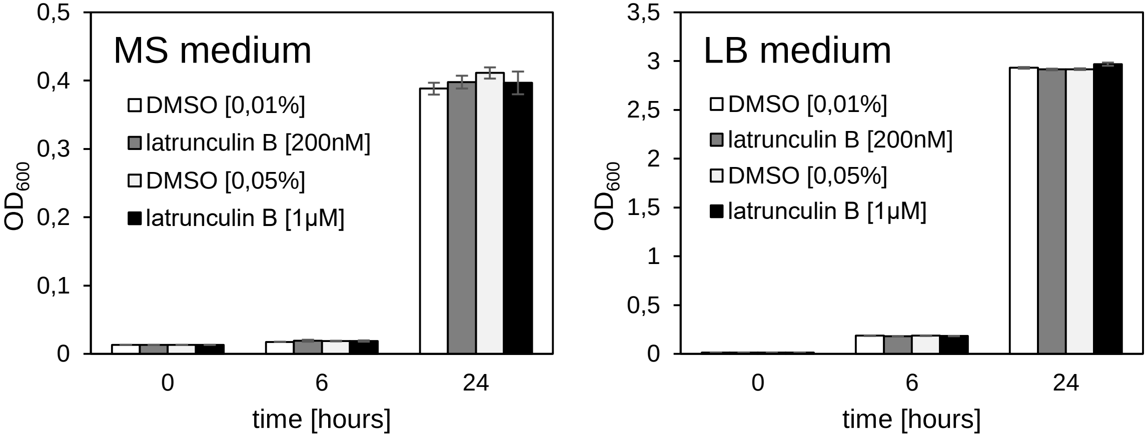

Fig. S4. Cytoskeletal drugs have no effect on the growth of *Pst* DC3000 *in vitro*.
Growth of *Pst* DC3000 in LB medium and MS medium. The media contained 0.2, 1 or 10 µM latrunculin B. As controls, 0.01, 0.05 or 0.5% DMSO was used. The values represent mean and error bars (SEM) from four independent samples.

FIGURE S6

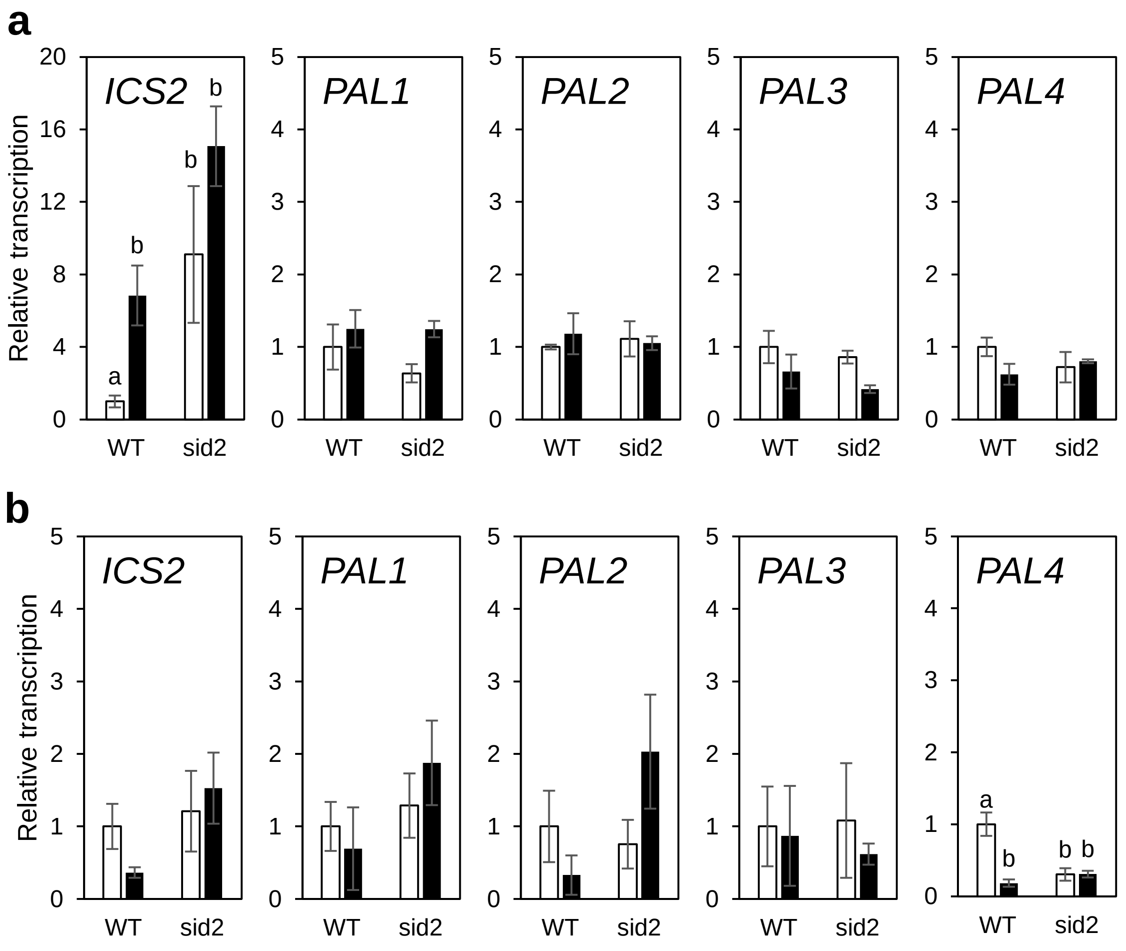

Fig. S6 Transcriptional analysis of SA biosynthetic genes after latrunculin B treatment. Seedlings were grown *in vitro* in liquid MS medium (a) and adult plants grown in soil for 4 weeks (b). Seedlings were treated for 24 h with 200 nM latrunculin B (latB) or 0.01% DMSO (control). 4-week-old plants were treated for 24 h with 1 µM latrunculin B (latB) or 0.05% DMSO (control). Transcription of SA biosynthetic genes *ICS2, PAL1, PAL2, PAL3* and *PAL4.* The transcription level was normalized to the reference genes, *SAND* (seedlings) *and TIP41* (4 week old plants). The values represent mean and error bars (SEM) from four independent samples. Statistical differences between the samples were assessed using a one-way ANOVA, with a Tukey honestly significant difference (HSD) multiple mean comparison post hoc test. Different letters indicate a significant difference, Tukey HSD, $P < 0.05$, n=4.

FIGURE S7

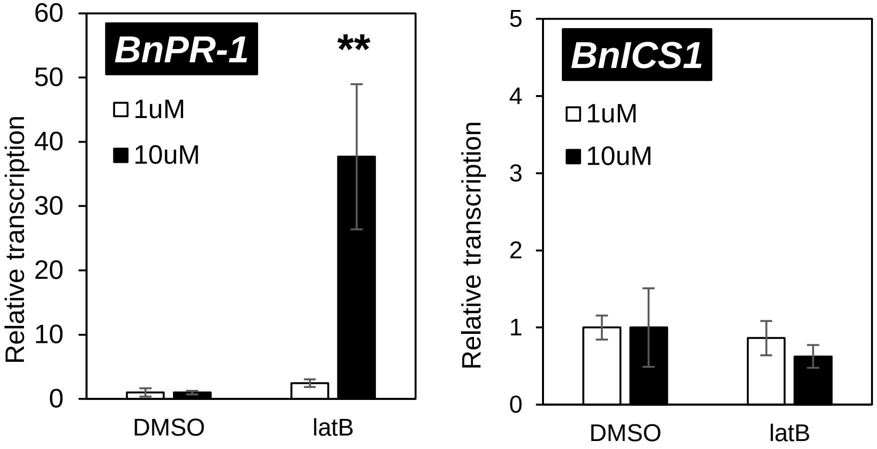

Fig. S7 Transcription of *BnPR-1* and *BnICS1* 72 h after latrunculin B treatment in *Brassica napus*. Cotyledons were treated for 72 h with infiltrations of 0.2, 1 or 10 µM latrunculin B (latB). Control cotyledons were treated for 72 h with a corresponding concentration of DMSO (0.05 or 0.5%). The transcription level was normalized to the reference gene, *BnTIP41*. The asterisks represent statistically significant changes in the latB-treated samples compared with controls (** P<0.01; two tailed Student´s t-test).

FIGURE S8

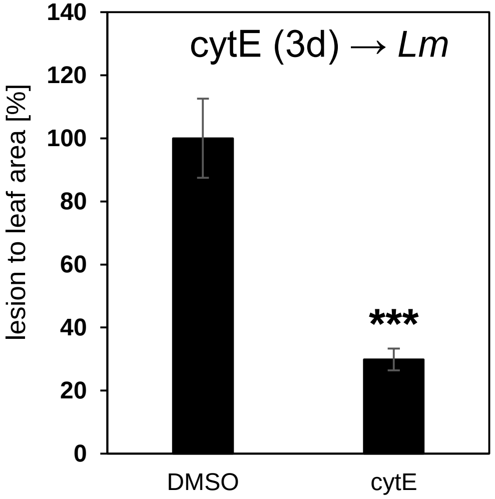

Fig. S5. Cytochalasin E induces resistance of *B. napus* to *L. maculans*.
Plant resistance was evaluated as relative lesion area (ratio of lesion area to whole leaf area) on the cotyledons. Cotyledons were treated with 10 µM cytochalasin E (cytE) or 0.25% DMSO (control) 3 days before inoculation with *L. maculans*. Lesions of DMSO controls in each treatment conditions were set as 100%. Data correspond to means ± SEM. The asterisks represent statistically significant changes in the cytE-treated samples compared with controls (*** $P<0.001$; two tailed Student´s t-test).

FIGURE S9

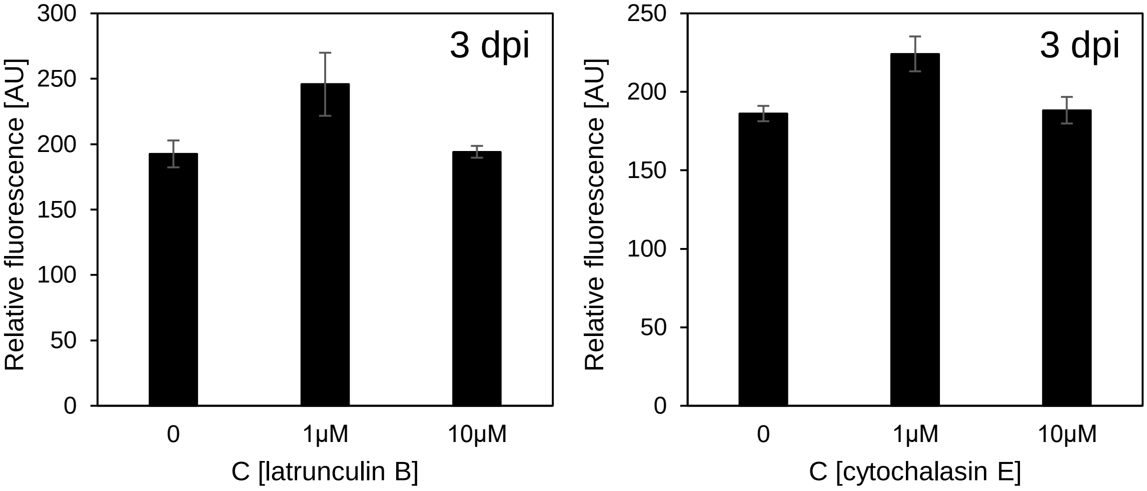

Fig. S6. Cytoskeletal drugs have no effect on the growth of *L. maculans in vitro*.
Growth of GFP-tagged *L. maculans* conidia in Gamborg medium supplemented with 1 or 10 μM latrunculin B, 1 or 10 μM cytochalasin E or 0.5% DMSO measured at 3 dpi. The values represent mean and error bars (SEM) from eight independent samples. Based on the performed t-test data are not statisticaly significant differented.

Table S1 Concentrations of phytohormones in *A. thaliana* seedlings

	DMSO MEAN [pmol/g(FW)]	DMSO SE	200 nM latrunculin B MEAN [pmol/g(FW)]	200 nM latrunculin B SE	TTEST (p value)
ABA	4.2	0.53	4.3	0.36	0.85
DPA	8.6	2.4	11.8	0.55	0.23
PA	0.66	0.49	0.96	0.31	0.61
ABA-GE	3.7	1.3	2.9	2.5	0.80
9OH-ABA	0.13	0.07	0.12	0.08	0.98
IAA	43.2	6.2	35.1	2.5	0.27
IAA-Asp	12.4	1.1	13.0	0.55	0.69
IA-GLU	21.2	0.62	20.6	1.8	0.76
OxIAA	169	23.0	197	19.9	0.39
OxIAA-GE	986	273	760	106	0.47
IAA-GE	17.8	1.2	14.4	2.3	0.24
PAA	27.2	1.4	27.9	2.0	0.79
IAM	2.9	0.74	0.89	0.35	0.047
IAN	19264	2169	18588	2301	0.84
SA	154	28.6	1109	153	0.0008
BzA	488	37.8	496	45.8	0.91
JA	112	28.1	253	28.3	0.012
JA-Ileu	20.3	7.0	32.4	7.8	0.29
cisOPDA	140	26.8	164	18.2	0.49
GA19	1.1	0.36	0.49	0.22	0.18

Abbreviations: IAA = indole-3-acetic acid; IAA-GE = IAA-glucose ester; IAA-Ala = IAA-alanine; IAA-Asp = IAA-aspartate; IAA-Glu = IAA-glutamate; IAA-Leu = IAA-leucine; IAA-Trp = IAA-tryptophane; IAA-Val = IAA-valine; IAA-PHE = IAA-Phenylalanine; OxIAA = oxo-IAA; OxIAA-GE = oxo-IAA-glucose ester; IAM = Indole-3-acetamide; IAN = Indole-3-acetonitrile (IAA precursor); 4Cl-IAA = 4-chloro-IAA; IPyA = indolepyruvic acid; 2,4-D = 2,4 dichlorophenoxyacetic acid; 2,4,5-T = 2,4,5 trichlorophenoxyacetic acid; IBA = indole-3-butyric acid; NAA = 1-Naphtaleneacetic acid; ABA = abscisic acid; ABA-GE = ABA-glucose ester; PA = phaseic acid; DPA = dihydrophaseic acid; 7OH-ABA = 7-hydroxy-ABA; 9OH-ABA = 9-hydroxy-ABA; NeoPA = Neophaseic acid; SA = salicylic acid; BzA = Benzoic acid; PAA = Phenylacetic acid; GAn = Gibberellin n=1,3,4,7,8,19,20,29; Strigol; JA = jasmonic acid; JA-Ile = JA-isoleucine; cisOPDA (JA precursor).

Table S2 List of primers used for qPCR

GENE	ACCESSION		SEQUENCE (5´ to 3´)	REFERENCE
AtSAND	AT2G28390	FP	CTGTCTTCTCATCTCTTGTC	1
		RP	TCTTGCAATATGGTTCCTG	
AtTIP41	AT4G34270	FP	GTGAAAACTGTTGGAGAGAAGCAA	2
		RP	TCAACTGGATACCCTTTCGCA	
AtPR-1	AT2G14610	FP	AGTTGTTTGGAGAAAGTCAG	1
		RP	GTTCACATAATTCCCACGA	
AtPR-2	AT3G57260	FP	TATAGCCACTGACACCAC	3
		RP	GCCAAGAAACCTATCACTG	
AtICS1	AT1G74710	FP	GCAAGAATCATGTTCCTACC	1
		RP	AATTATCCTGCTGTTACGAG	
AtICS2	AT1G18870	FP	TGTCTTCAAAGTCTCCTCTG	1
		RP	CTTCCTCCAAACTCATCAAAC	
AtPAL1	AT2G37040	FP	TAGTAGTGATTGGGTTATGGAG	1
		RP	GTGGCTTGTTTCTTTCGT	
AtPAL2	AT3G53260	FP	AGCAGTGATTGGGTTATGG	1
		RP	ATGTCTCCTTCGTGTTTCC	
AtPAL3	AT5G04230	FP	GTGTGAAGGCGAGTAGTG	1
		RP	GAAGCGTGTTTGACCTGT	
AtPAL4	AT3G10340	FP	GCTCAACTTCTCCACGAAAT	1
		RP	CGACACATCTATCAAAGGGTT	
BnTIP41		FP	AGAGTCATGCCAAGTTCATGGTT	4
		RP	CCTCATAAGCACACCATCAACTCTAA	
BnPR-1		FP	CATCCCTCGAAAGCTCAAGAC	5
		RP	CCACTGCACGGGACCTAC	
BnICS1		FP	CAAACTCATCATCTTCCCTC	5
		RP	AGCGTGACTTACTAACCAG	

1 Sasek, V. *et al.* Constitutive salicylic acid accumulation in pi4kIIIbeta1beta2 Arabidopsis plants stunts rosette but not root growth. *New Phytol* **203**, 805-816, doi:10.1111/nph.12822 (2014).

2 Czechowski, T., Stitt, M., Altmann, T., Udvardi, M. K. & Scheible, W. R. Genome-wide identification and testing of superior reference genes for transcript normalization in Arabidopsis. *Plant Physiol* **139**, 5-17, doi:10.1104/pp.105.063743 (2005).

3 Matouskova, J. *et al.* Changes in actin dynamics are involved in salicylic acid signaling pathway. *Plant Sci* **223**, 36-44, doi:10.1016/j.plantsci.2014.03.002 (2014).

4 Chen, X., Truksa, M., Shah, S. & Weselake, R. J. A survey of quantitative real-time polymerase chain reaction internal reference genes for expression studies in Brassica napus. *Anal Biochem* **405**, 138-140, doi:10.1016/j.ab.2010.05.032 (2010).

5 Sasek, V. *et al.* Recognition of avirulence gene AvrLm1 from hemibiotrophic ascomycete Leptosphaeria maculans triggers salicylic acid and ethylene signaling in Brassica napus. *Mo[illegible] Plant Microbe Interact* **25**, 1238-1250, doi:10.1094/MPMI-02-12-0033-R (2012).
